# Uncovering Flat and Hierarchical Topics by Community Discovery on Word Co-occurrence Network

**DOI:** 10.1007/s41019-023-00239-2

**Published:** 2024-03-13

**Authors:** Eric Austin, Shraddha Makwana, Amine Trabelsi, Christine Largeron, Osmar R. Zaïane

**Affiliations:** 1 University of Alberta, Edmonton, AB T6G 2R3 Canada; 2 Alberta Machine Intelligence Institute, Edmonton, AB T5J 3B1 Canada; 3 Universitè de Sherbrooke, Sherbrooke, QC J1K 2R1 Canada; 4 Hubert Curien Laboratory, Universitè Jean Monnet, Saint-Etienne, France

**Keywords:** Topic modeling, Community mining, Hierarchical topics, Information networks, Graphs, Natural language processing, Data mining

## Abstract

Topic modeling aims to discover latent themes in collections of text documents. It has various applications across fields such as sociology, opinion analysis, and media studies. In such areas, it is essential to have easily interpretable, diverse, and coherent topics. An efficient topic modeling technique should accurately identify flat and hierarchical topics, especially useful in disciplines where topics can be logically arranged into a tree format. In this paper, we propose Community Topic, a novel algorithm that exploits word co-occurrence networks to mine communities and produces topics. We also evaluate the proposed approach using several metrics and compare it with usual baselines, confirming its good performances. Community Topic enables quick identification of flat topics and topic hierarchy, facilitating the on-demand exploration of sub- and super-topics. It also obtains good results on datasets in different languages.

## Introduction

Topic modeling discovers the themes of collections of unstructured text documents [36, 46, 79]. Topics can act as features for document classification and indices for information retrieval. However, one of the most important functions of these topics is to assist in the exploration of large corpora. Researchers in all fields and domains seek to better understand the main ideas and themes of document collections too large for a human to read and summarize. This requires topics that are interpretable and coherent to human users.

Interpretability is a necessary but not sufficient condition for a good topic model. Topics naturally exist in a hierarchy. There are larger, more general super-topics and smaller, more specific subtopics. “Sports” is a valid topic in that it represents a concept. “Football” and “Olympics” are also topics. They are not completely distinct from “Sports” but rather are sub-topics that fall within sports, i.e., they are child topics of the “Sports” parent topic in the topic hierarchy. Topics also relate to each other to varying degrees. The “movie” topic is more similar to the “television” topic than the “food” topic. This relationship structure is also key to understanding the topical content of a corpus. Topic modeling methods that simply provide the user with a set of topics are not as useful and informative as those that can provide this hierarchy and structure.

When detecting and organizing the topics, diversity is crucial to avoid having several topics that are basically the same and thus preventing redundancy in the extracted topics. Having a variety of topics also enables a more thorough and nuanced comprehension of the corpus. Let’s imagine we utilize topic modeling to identify the major themes in a corpus of news articles regarding the economy. Without topic diversity, we might end up with multiple topics that are essentially the same, such as “jobs" and “employment." However, with topic diversity, we might also identify topics such as “tax policy," “trade agreements," and “consumer spending," which provide a more diverse and nuanced understanding of the economy beyond just the labor market.

The capability of topic modeling to accommodate multiple languages is another crucial component. This ability is very useful when analyzing text corpora from geographical areas with several official languages or social media data from various communities. Topic modeling supporting different languages can also help researchers who need to analyze enormous volumes of data quickly on common computer hardware.

Recently, a new domain has emerged where topics can provide utility: conversational agents, which are computer programs that can carry on a human-level conversation. The conversation is an end in itself; the purpose of speaking with a conversational agent is to converse, to be entertained, to express emotion and be supported. The awareness and use of the topics of discussion are key abilities that an agent must possess to be able to carry on a conversation with a human. Previous work has used the detected topic of conversation to enrich a conversational agent’s responses [21]. However, more can be done with topics to improve the abilities of a conversational agent given the right topic model that provides a topic hierarchy and structure. It can be used to detect and control topic drift in the conversation so that the agent’s responses make sense in context. If the user is engaged with the current topic, then the agent can stay on topic or detect sub-topics to focus the conversation. The agent can detect super-topics to broaden the range of conversation. The agent should be able to move to related topics or, if the user becomes bored or displeased, jump to dissimilar topics. This type of control over the flow of the conversation is crucial to human communication and is needed for human-computer interaction as well.

In the literature, various models have been proposed to automatically discover topics in collections of text documents. The most widely used topic model, Latent Dirichlet Allocation (LDA), only provides a simple set of topics without a hierarchy or structure and it has other drawbacks. The number of topics must be specified, requiring multiple runs with different numbers of topics to find the best topics. It performs poorly on short documents. Moreover it is not deterministic. Thus, different runs on the same corpus can produce different topics, especially if the order of the documents is different [48]. Finally, common terms can appear in many different topics, reducing the uniqueness of topics [57].

Neural networks have pushed forward the state-of-the-art in topic modeling. A relatively new algorithm called Top2Vec [2] uses word embeddings but suffers from topic overlap [23]. Another embedding-based approach, BERTopic [31], requires specialized hardware. Both Top2Vec and BERTopic are suitable for short-text data analysis [22, 69]. Neural topic models, such as nTSNTM [15], produce more coherent topics than LDA but retain many of its weaknesses, such as the need to specify the number of topics and the tendency to find models with many redundant topics [12]. These models also require more computational resources and specialized hardware. Hierarchical topic models, such as Hierarchical LDA (HLDA) [29], Pachinko Allocation Model (PAM) [45], and Hierarchical Pachinko Allocation (HPA) [55], have not demonstrated good hierarchical relationships in terms of topic specialization and affinity between super and subtopics.

Thus, although neural topic models have produced topics of greater coherence, they retain many of the weaknesses of LDA, such as the need to specify the number of topics, while having a tendency to find redundant topics [12] and demanding greater computational resources and specialized hardware.

These drawbacks have inspired us to search for a new approach to topic modeling. We desire a method that can operate quickly on commodity hardware and that deterministically provides not only a set of topics but their relationships and a hierarchical structure. It should also supports different languages while maintaining topic diversity and interpretability. Given these expectations, it seems natural to take an information network-based approach.

Our topic modeling algorithm, Community Topic (CT), mines communities from networks constructed from term co-occurrences. These topics are collections of vocabulary terms and are thus easily interpretable by humans. The fractal nature of the network representation provides a natural topic hierarchy and structure. The topic hyper-vertices form a network with connections of varying strength between the topic vertices derived from the aggregated edges between their constituent word vertices. Super-topics can be mined from this topic network. Indeed, each topic itself is also a sub-graph with regions of varying density of connections that can be mined to find sub-topics. Our algorithm has only a single hyperparameter and can run quickly on simple hardware which makes it ideal for researchers from all fields for exploring a document collection. With proper data pre-processing, this algorithm is also language-agnostic, enabling it to be applied to diverse linguistic datasets.

In this paper, Sect. 1 presents a review of the current state-of-the-art in topic modeling. Section 2 describes our algorithm, how it constructs term co-occurrence networks and mines topics from them. It explains how our method discovers topic hierarchies and can adapt on-the-fly based on user requirements. To assess our algorithm’s effectiveness, we evaluated it both for simple and hierarchical topic discovery, and for different languages. Our evaluation metrics include coherence, interpretability, diversity, hierarchical specialization, and affinity. Our experimental results, presented in Sect. 5 after our evaluation protocol detailed in Sect. 4, demonstrate that our approach outperforms existing methods in finding a more coherent topic structure and establishing a stronger relationship between parent and child topics. Thus, our algorithm yields flat or hierarchical topics efficiently and enables on-demand sub- and super-topic discovery. It should be noted that the open-sourced python library along with code and usage tutorial is available online.^1^

## Related Work

Topic modeling emerged from the field of information retrieval and research to more effectively represent documents for indexing, query matching, and document classification. The performance of topic models on these tasks has been surpassed by deep neural models but topic models have become extremely popular tools of applied research both inside and outside of computing science [34]. For a good overview of the subject, we refer the reader to the recent survey of Churchill and Singh [16].

### Early Approaches

One early approach is Latent Semantic Analysis (LSA) [18] which decomposes the term-by-document matrix to find vectors representing the latent semantic structure of the corpus and can be viewed as (uninterpretable) topics that relate terms and documents. Another matrix decomposition method is Non-negative Matrix Factorization [44]. Researchers unsatisfied with the lack of a solid statistical foundation to LSA developed Probabilistic Latent Semantic Analysis (pLSA) [33] which posits a generative probabilistic model of the data with the topics as the latent variables. A drawback of pLSA is that the topic mixture is estimated separately for each document. Latent Dirichlet Allocation (LDA) [7], not to be confused with Linear Discriminant Analysis, was developed to remedy this. LDA is a fully generative model as it places a Dirichlet prior on the latent topic mixture of a document. The probability of a topic *z* given a document *d*, $p(z|d;\theta )$, is a multinomial distribution over the topics parameterized by $\theta$ where $\theta$ is itself a random variable sampled from the prior Dirichlet distribution. The number of topics must be specified and the model provides no topic hierarchy or structure.

There have been many methods developed that attempt to improve upon LDA. Promoting named entities to become the most frequent terms in the document has been tried [40]. In [89], the authors use a process to identify and re-weight words that are topic-indiscriminate. To improve the performance of LDA on tweets, the authors of [52] pool tweets into longer documents. Supervised LDA (sLDA) is an LDA extension that incorporates supervised information such as class labels [51]. In the same vein, the MetaLDA model [93] incorporates also meta information such as document labels. Structural Topic Models (STM) [67] is an LDA extension that models the structure of the covariates and their relation to topics while Relational Topic Models (RTM) models co-occurrence patterns between documents [13]. The author-topic model [73] extends LDA by conditioning the topic mixture on document author and, the Correlated Topic Model (CTM) [5] takes into account the correlations between topics but its computational cost may limit its scalability. Finally, the Dynamic Topic Model [6] allows for the modeling of topic evolution over time.

### Hierarchical Topic Detection

Topic modeling algorithms like LDA [7] or pLSA [33] are not designed to detect topic hierarchies. They are only able to capture correlations among words but not over the topics due to the fact that the topics in the documents share a common distribution, usually a Dirichlet distribution. To overcome this limitation, it is necessary to model the distribution of the hierarchy of topics; which can be done using the nested Chinese restaurant process (nCRP) [8, 29] or the nested hierarchical Dirichlet process (nHDP) [62]. Likewise, several hierarchical methods have been developed to find super and sub-topics in documents. The Hierarchical LDA model (HLDA) [29] models the topic hierarchy using a tree structure. The depth of the tree must be specified but the number of topics is discovered. Another flexible generalization of LDA is the Pachinko Allocation Model (PAM) [45]. Like HLDA, PAM allows for a hierachy of topics but this hierarchy is represented by a directed acyclic graph rather than a tree of fixed depth, allowing for a variety of relationships between topics and terms in the hierarchy, although this structure must be specified by the user. Besides these two important representatives of hierarchical topic models, there are also their derived versions such as (HLLDA) [63] or (HPA) [55]. Hierarchical Labeled-LDA (HLLDA) introduced label prior in HLDA whereas Hierarchical Pachinko Allocation (HPA) [55] extends PAM to generate a hierarchy of medoids, useful for identifying global and local structures in the data. However, HPA can be computationally expensive and requires hyperparameter tuning. For more details on these hierarchical models and their variants, we refer the reader to the survey of Liu et al. [47]. However, we could notice that, in addition to the parameterization and the computational cost inherent in this family of models, hierarchical type models have other disadvantages, such as topic incoherence, unreasonable hierarchical structure, and issues related to the depth of the hierarchy, as pointed out by [80].

### Multilingualism and Short Documents

Although many of the topic models discussed above have been successful in analyzing documents, their applicability to different languages remains unclear. Multilingual topic models (MTMs) have been proposed to overcome this limitation by uncovering latent topics across languages and revealing commonalities and differences across cultures [61, 71]. In a recent study [90], Yang et *al.* improved upon previous MTMs by learning weighted topic links and connecting cross-lingual topics only when the dominant words defining them are similar, resulting in better classification performance than LDA and previous MTMs.

Another important aspect of topic modeling is its application to short documents. To address this, various methods have been proposed, such as Sentence-LDA [66], which models topics at the sentence-level, and Dirichlet Multinomial Mixture Model (DMM) [91], Biterm topic model [88], and Dirichlet Process Multinomial Mixture Model (DPMM) [66], which are specifically designed for short text topic modeling.

### Recent Approaches

In recent years, new topic models have emerged based on neural networks [83]. For instance, the Embedded Topic Model (ETM) [19] combines word embeddings trained using the continuous Skip-gram algorithm [54] with the LDA probabilistic generative model. Another approach is to use a variational autoencoder (VAE) [38, 39] to learn the probability distributions of a generative probabilistic model, as with the neural variational document model (NVDM) [53], the stick-breaking variational autoencoder (SB-VAE) [56], ProdLDA [72], and Dirichlet-VAE [12]. These models discover topics that are qualitatively different than those found by traditional LDA, although there is debate as to whether they are truly superior [34]. Other approaches use word embeddings learned by a neural network but do not use the probabilistic generative model framework. For example, the Top2Vec algorithm [2] clusters document vectors learned by the Doc2vec algorithm [43]. Correlation Explanation (CorEx) is another topic model that produces informative topics about a set of documents [27]. However, it may face difficulties in accurately identifying topics in datasets where words are generated by multiple topics or where topics have overlapping words. In this family, we can also mention BERTopic, an unsupervised method that does not require the number of topics to be specified a priori [31]. It uses pre-trained BERT embeddings but may not perform as well on domain-specific or low-resource datasets where pre-training may be limited.

Neural models that provide a topic hierarchy have also been developed. In [92], the authors develop Weibull hybrid autoencoding inference (WHAI) to model multiple layers of priors for deep LDA and thus multiple layers in a topic hierarchy. However, the number of hyperparameters, complicated training process, and need for special hardware make this type of model unsuitable for applied researchers seeking a tool for corpus exploration. TSNTM [35], nTSNTM [15] are two other models designed to detect topic hierarchies. They exploit a doubly-recurrent neural network (DRNN) to parameterize the topic distribution over an infinite tree. Based on the same principle, HTV [64] is a neural topic model designed for jointly detecting topic hierarchies and visualization. The identification of subtopics was also addressed by embedding the words and topics in the same vector space, of the Euclidean type for SawETM [20], an extension of ETM [19], or hyperbolic for [87]. However as highlighted by Wu et al [86], the topic hierarchy cannot grow dynamically since their layers must be fixed before training. Moreover, it should be noted that although these models have achieved high coherence scores, they are also computationally expensive and require tuning of many hyperparameters.

Finally, among all the models in the literature, the one that is closest to ours is hSBM [28] since it also discovers topics by looking for communities in network. But, unlike CT, hSBM detects communities using a stochastic block model (SBM) and therefore, as the probabilistic topic models previously mentioned, it suffers from the same shortcomings that led us to propose our model Community Topic (CT), described in the next section.

## Community Topic

Community Topic (CT) is a topic modeling algorithm that leverages community detection to identify topics in a given corpus. CT is based on the assumption that words which are used or which occur in a same sentence or a same sequence of words, are more likely to relate to the same topic. This underlying assumption is justified by the works of Harris (1954) on the distributional structure who states that it is possible to define a linguistic structure solely in terms of patterns of co-occurrences of its elements [32]. It is also on this assumption that most current language embedding models, such as Word2Vec, are based [54]. Indeed, they rely on the hypothesis that semantically similar words co-occurs in the documents and thus they should be close to each others in the embedding space.

CT supports both flat and hierarchical topic modeling and the code is available in an open-sourced library^2^ with a tutorial.^3^

CT follows several steps to identify topics in the corpus as discussed in below subsections, just after a brief reminder of notions from social network analysis, useful in the sequel.

### Network and Communities

A comprehensive review of network theory is beyond the scope of this paper and we refer the reader to [58, 84] for more details. We just define sufficient terminology to be able to understand our method.

A network is represented by a graph $G = (V, E)$ where *V* is the set of vertices and *E* is the set of edges. A network may be **unweighted**, in which case there is a binary alternative between the existence or non-existence of an edge $e_{i,j}$ between any two vertices $v_i, v_j \in V$ that indicates a relationship between those vertices. A network may be **weighted**, in which case an edge $e_{i,j}$ has an associated weight $w_{i,j}$ which is a numeric value that characterizes in some way the relationship between vertices $v_i$ and $v_j$. The **degree** of a vertex $v_i$, denoted $k_i$, is the number of edges connected to that vertex, i.e., $k_i = |\{e_{i,j}: v_j \in V\}|$. The **internal degree** of a vertex $v_i$, denoted $k^{int}_i$, is the number of edges that connect $v_i$ to another vertex of the same community. The **weighted degree** of a vertex $v_i$, denoted $k^w_i$, is the sum of the weights of all edges connected to that vertex, i.e., $k^w_i = \sum _{v_j \in V}w_{i,j}$. The **internal weighted degree** of a vertex $v_i$, denoted $k^{w,int}_i$, is the sum of the weights of all edges that connect $v_i$ to another vertex of the same community. The **embeddedness** of a vertex $v_i$ is $k^{int}_i / k_i$. The **weighted embeddedness** of a vertex $v_i$ is $k^{w,int}_i / k^w_i$.

Community structure is the tendency of networks to consist of groups of vertices where the density of edges within the group is much higher than the density of edges between groups. These groups of highly-connected vertices are called communities. There is no single formal accepted definition of a community or how dense the connections must be to form a community. Certainly a fully connected group of vertices, i.e., a clique, would constitute a community, but communities need not be so densely connected. We are interested in finding all of the communities of the network. This global partitioning of the network into communities is called **community detection**. Many different community detection algorithms have been developed over the years and are reviewed in [17, 25, 26, 74].

Our community detection-based topic modeling algorithm Community Topic (CT) has three main steps. First, a network is constructed from the document corpus. After the network is constructed, CT applies a community detection algorithm to find the communities in the network. Finally, the communities are filtered out and, each topic (i.e., community) is sorted so that the most important and relevant terms for the topic come first and the topics are returned. By this way, CT can identify both flat topics within a corpus but by adding a fourth step it can also discover hierarchical topics. These different steps are detailed below and, the pseudo-codes for each type of topic modeling are given in Algorithm 1 for flat topics and in Algorithm 2 for hierarchical topics.

### Co-occurrence Network Construction

First, a network is constructed from the document corpus with terms as vertices. An edge exists between a pair of vertices $v_i$ and $v_j$ if the terms $t_i$ and $t_j$ co-occur in the same sentence or within a sliding window applied on the text. The weights of edges are derived from the frequency of co-occurrence. One method is to use the raw count as the edge weight. However, this does not adjust for the frequency of the terms themselves so more common terms will tend to have higher edge weights. An alternative weighting scheme is to use normalized pointwise mutual information (NPMI) between terms (Eq. 1).

1 $$\begin{aligned} NPMI(t_i, t_j) = \frac{log\frac{p(t_i, t_j)}{p(t_i)p(t_j)}}{-log(p(t_i, t_j))} \end{aligned}$$

NPMI assigns higher values to pairs of terms $t_i$ and $t_j$ whose co-occurrence, $p(t_i, t_j)$, is more frequent than what would be expected if their occurrences in the texts were random, $p(t_i)p(t_j)$. This is normalized to adjust for the frequencies of the terms in the corpus. The edges of the network are thresholded at 0, i.e., those edges with weights less than or equal to 0 are removed from the network. This is because the community mining algorithm we will use to discover topics uses modularity *Q* [59] to discover the more densely connected regions of the network. This formula uses the product of the weighted degrees of two vertices to determine the expected value of the strength of their connection if the graph was random, which does not work if a vertex has a negative weighted degree.

2 $$\begin{aligned} Q = \frac{1}{2m}\sum _{ij}\left( A_{i,j} - \frac{k^w_i k^w_j}{2m}\right) \delta (C_i, C_j) \end{aligned}$$

Here *m* is the sum of weights of all edges in the network, $A_{i,j}$ is the weight of the edge connecting $v_i$ and $v_j$, $k^w_i$ ($k^w_j$) is the sum of weights of edges incident to $v_i$ ($v_j$), $C_i$ ($C_j$) is the assigned community of $v_i$ ($v_j$), and $\delta$ is an indicator function that returns 1 when the two arguments are equal and 0 otherwise.

The distribution of edge weights differs greatly between the raw count and NPMI. The raw count weights follow a power law distribution with the vast majority of edges having very low weight and very few edges with very high weight. This mirrors the power law distribution of term frequencies. Given this distribution of term frequencies, a given edge weight value can carry very different information. An edge weight of 2 could indicate a significant relationship between two terms that occur 5 times each. Between two terms that occur hundreds of times each, an edge weigh of 2 would be noise. When we convert the edge weights to NPMI values, they are scaled to the range [−1,+1] and high values are assigned to edges that represent frequent co-occurrence relative to the frequencies of the connected terms. This distribution resembles a bell curve. We see very few edge weights less than or equal to 0 that will be removed by thresholding. This indicates that conditioned on co-occurring at least once, two terms are likely to co-occur more often than would be expected by chance. In our experiments we found slightly better results using the NPMI edge weights.

### Community Mining

Once the co-occurrence network is constructed, CT discovers topics by applying a community detection method.

**Fig. 1:**
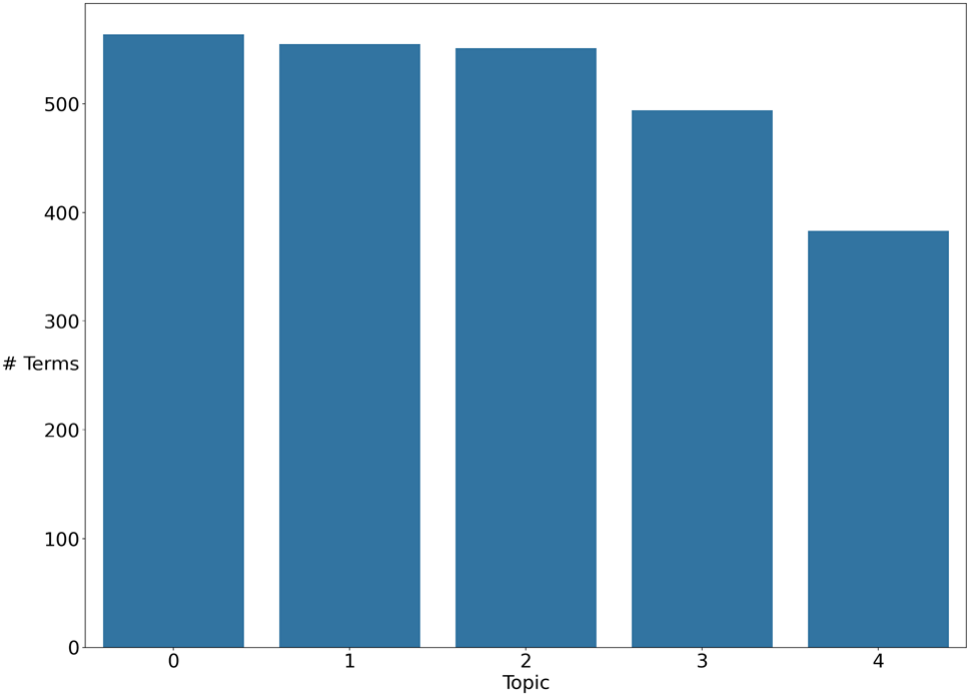
Distribution of community sizes found by Leiden with resolution parameter 1.0 on BBC News dataset

**Fig. 2:**
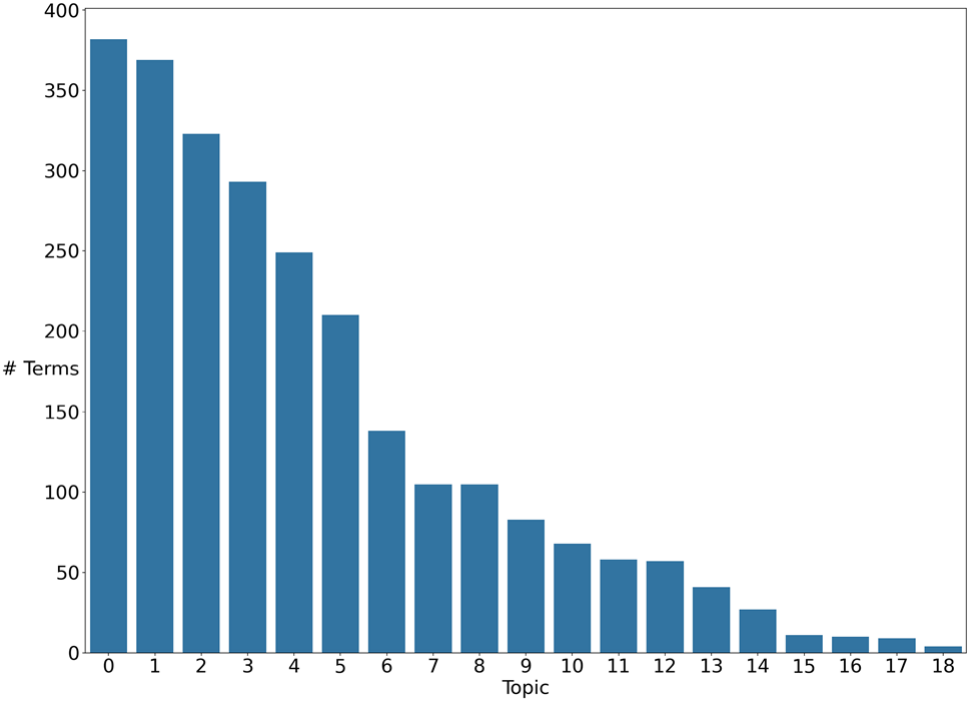
Distribution of community sizes found by Leiden with resolution parameter 1.5 on BBC News dataset

A community is a group of vertices that have a greater density of connections among themselves than they do to vertices outside the group. Many community detection algoritms exist and have been surveryed in other papers such as [17, 25, 26] or [74]. CT employs the Leiden algorithm [77] as this was found to work best in experimentation but other algorithms can be used. The Leiden algorithm has a resolution parameter that is used to set the scale at which communities are discovered. Smaller values of this parameter lead to larger communities being found and larger values lead to smaller communities. For illustration, Fig. 1 shows the distribution of community sizes found when using a Leiden resolution parameter of 1.0 on the BBC News dataset.^4^ CT returns 5 large topics that correspond to the five article categories of the dataset. In Fig. 2, we see that a resolution parameter of 1.5 returns a greater number of small topics with a greater variance of topic size, from hundreds of terms to just a few. This represents the only hyperparameter necessary for CT and is less a value that needs to be carefully tuned for good performance but is rather a way for the user to get communities of a desired size. However, other community detection algorithms can be used instead of Leiden, such as Louvain [9] which does not require a parameter, it is easy to make CT free parameter. It should however be noted that the choice of the community detection algorithm can impact the topics and topic quality. In our experiments, we retained Leiden since it has shown better performances than Louvain [77], itself being in general better than Girvan–Newman [9].

**Algorithm 1:**
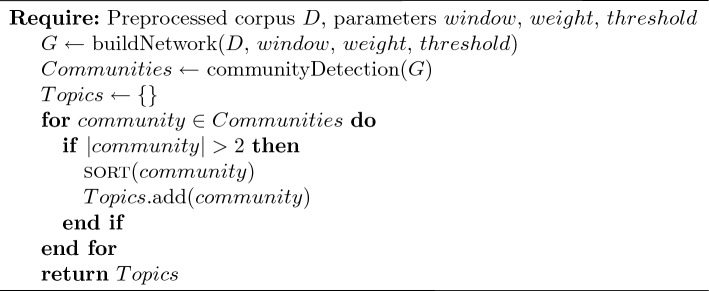
Flat Community Topic

### Topic Filtering and Term Ordering

Once the communities are discovered, small communities of size 2 or less are removed as outliers. Probabilistic graphical topic models such as LDA produce topics that are probability distributions over vocabulary terms. The most important terms for a topic are simply those that have the highest probabilities. The communities discovered by the Leiden algorithm are sets of vertices, so CT needs a way of ranking the terms represented by those vertices. To do so, we take advantage of the graph representation and use internal weighted degree to rank vertices/terms, which is calculated as the sum of weights of edges incident to a vertex that connect to another vertex in the same community/topic. This gives higher values to terms that connect strongly to many terms in the same topic and are thus most representative of that topic. Once the filtering and ordering is complete, the set of topics is returned to the user.

### Topic Hierarchy

This basic formulation of CT produces a set of topics like vanilla LDA. However, there exists a natural structure to the graph representation and it is straightforward to adapt CT to return a hierarchy. By taking advantage of the community structure generated on the graph or the community detection algorithm, it is possible very simply to obtain a hierarchy of topics. There are two ways to do this. Firstly, by iteratively applying community detection to each topic sub-graph, CT discovers the next level of the topic hierarchy. This can be done to a specified depth or we can allow CT to uncover the entire hierarchy by stopping the growth of the topic tree once the produced sub-topics are smaller than three terms. An example of 3 levels of topics discovered on the BBC corpus is show in Fig. 6. The level 1 topics correspond to the 5 article categories of the corpus. Level 2 (in green) and then 3 (in orange) show increasingly specific sub-topics. These levels 2 and 3 were respectively obtained by applying CT to the subgraphs associated with the topics "Business" and "Tech" and then "Web".

If a low Leiden resolution parameter is initially used, CT produces many small topics i.e., communities in the first partition. Applying the iterative community detection process to the network of topic vertices groups these small sub-topics into super-topics. We can see an example of this in Fig. 7 that shows the clustering of the initial small topics discovered on the BBC corpus into super-topics which roughly correspond to the 5 article categories of the corpus. The topic hierarchy can also be constructed in a bottom-up fashion. This amounts to exploiting the iterative nature of community detection algorithms like Leiden, which optimizes a quality function such as modularity in three elementary steps: (1) local moving of nodes; (2) refinement step and (3) aggregation of the network. In the local moving step, individual nodes are moved to the community that yields the largest increase in the quality function. In the aggregation step, an aggregate network is created based on the partition obtained in the local moving phase. Thus, each community in the partition obtained at the end of second step becomes a node in the aggregate network built in the third step. The three steps are repeated until the quality function cannot be increased further and at each iteration of these three steps a coarser partition is built leading to a hierarchy. For more detail on the iterative process of Leiden and the construction of the hierarchy, we refer the reader to [77].

Note that if a low Leiden resolution parameter is initially used, CT produces many small topics i.e., communities in the first partition. Applying the iterative community detection process to the network of topic vertices groups these small sub-topics into super-topics. We can see an example of this in Fig. 7 that shows the clustering of the initial small topics discovered on the BBC corpus into super-topics which roughly correspond to the 5 article categories of the corpus. The pseudocode of CT for discovering hierarchical topics is given in Algorithm 2.

**Algorithm 2:**
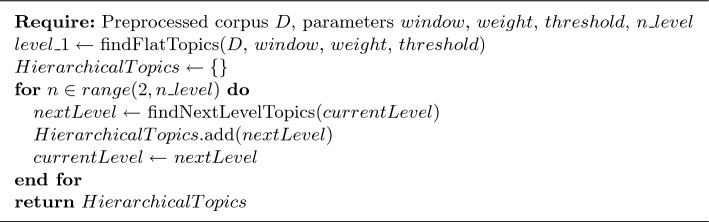
Hierarchical Community Topic

### CT Complexity

The complexity of CT depends on the size of the graph and the underlying community detection algorithm. As indicated in Sect. 4.2, a preprocessing of the documents not only allows to reduce the number of vertices of the graph but also leads to better results. As for the community detection algorithm, if we know that the optimization of modularity is NP-hard [11], it is also admitted in the literature that the convergence of a heuristic like Louvain is quasi-linear (in O(nlog(n) where n is the number of vertices) on real networks [3], making Louvain one of the most efficient community detection algorithms [41, 89], just after Leiden which is even faster [77].

## Evaluation Protocol

We extensively evaluate Community Topic through empirical experiments to identify the optimal hyperparameters and also compare CT with various baselines. Our experiments encompass flat topic modeling, hierarchical topic modeling, and analysis of different languages. All the data and code used in the experiments are publicly available on our GitHub repository.^5^

### Datasets

We use four datasets to assess the effectiveness of various topic modeling approaches, namely 20Newsgroups,^6^ Reuters21578,^7^ BBC News,^8^ and EuroParl.^9^ The 20Newsgroups dataset comprises 18,846 posts from the Usenet discussion forum covering 20 distinct topics such as "atheism" and “hockey". The Reuters21578 dataset consists of 21,578 financial articles that were published on the Reuters newswire in 1987 and cover economic and financial topics such as “grain" and “copper". The BBC News dataset comprises 2,225 articles grouped into five categories: “business", “entertainment", “politics", “sport", and “tech". The EuroParl parallel corpus is extracted from the transcripts of European Parliament proceedings. We have randomly selected 19,000 documents from EuroParl as the training dataset and 6,000 documents as the test dataset. This corpus includes versions in 21 European languages, and hence we have used this particular dataset to compare the performance of Community Topic and other baselines across multiple languages.

### Preprocessing

To prepare a text corpus for topic modeling, there are numerous techniques that have been found to be effective in the literature. We use spaCy^10^ to lowercase and tokenize the documents and to identify sentences, parts-of-speech (POS), and named entities. We employ the appropriate spaCy model depending on the language of the input dataset. Only noun-type entities, such as EVENT, FAC (buildings), GPE (geo-political entities), LOC (non-GPE locations), ORG (organizations), PERSON, PRODUCT, and WORK OF ART, are detected and merged into single tokens, for example, “united”, “states”, “of”, and “america” become “united states of america”.

While stemming and lemmatization have been commonly used in the topic modeling literature, the authors of [70] found that they do not improve topic quality and hurt model stability so we do not stem or lemmatize. We remove stopwords and terms that occur in over $90\%$ of documents. This formula is more effective in larger corpora but is only proportional to$\sqrt{|d|}$. Following [34], we remove terms that appear in fewer than $2(0.02|d|)^{1/log 10}$ documents. It was shown in [49] that topic models constructed from noun-only corpora were more coherent so we detect and tag parts-of-speech to be able to filter out non-noun terms as in [14]. This is intuitive as adjectives and verbs can be used in many different contexts, e.g., one can “play the piano”, “play baseball”, “play the stock market”, and “play with someone’s heart”, but music, sports, finance, and romance are separate topics. Even with nouns there are issues with polysemy, i.e., words with multiple meanings and thus multiple different common contexts. To help with this problem, we use Gensim^11^ using NPMI to extract meaningful *n*-grams [10]. An *n*-gram is a combination of *n* adjacent tokens into a single token so that a term such as “microsoft_windows” can be found and the computer operating system can be distinguished from the windows of a building. We apply two iterations so that longer *n*-grams such as “law_enforcement_agencies” can be found. To support different languages, we use connector words specific to each language. For English we use connector words from Gensim library and for other languages we translate these connector words into that language for consistency purpose. Currently, our pre-processing module supports five languages: English, Italian, French, German, and Spanish. We compare the quality of topics to ensure that different algorithms are not more sensitive to generic terms and that there are no topical adjectives or verbs with n-gram combinations.

### Hyperparameter Tuning

We performed extensive experiments on the four datasets mentioned above by training them with and without parts-of-speech filtering. Co-occurrence networks were created using both raw count and NPMI edge weights, with threshold values of 0 and 2 for count networks and 0 and 0.35 for NPMI networks. We used a sentence co-occurrence definition and sliding windows of size 5 and 10. Community detection was performed using WalkTrap [65] and Leiden [77] algorithms with resolution parameters of 1, 1.5, 2, and 2.5. The Leiden resolution parameter determines the scale of discovered communities, with larger values yielding more, smaller communities.

Topics were ordered by various metrics such as degree, weighted degree, internal degree, internal weighted degree, embeddedness, and weighted embeddedness. The results were evaluated with $C_{V}$ and $C_{NPMI}$, described in Sect. 4.4, with top-N values of 5, 10, and 20, leading to a total of 18,144 evaluations. Based on our results, we found that Community Topic works best with the Leiden algorithm. Since Leiden performed well on all datasets with the same set of hyperparameters, we recommend using a sentence co-occurrence window, NPMI edge weights, no thresholding, and noun-only POS filtering as the standard settings and report results corresponding to this setting. These hyperparameters are chosen such that the algorithm is hyperparameter-free, but our published library allows for flexibility in experimenting with different combinations.

### Evaluation Metrics

Different evaluation metrics can serve as objective targets to better analyze a topic model’s behavior [75]. The following metrics have been used in our experiments.

#### Topic Coherence Metrics

Even if perplexity is frequently considered for topic models evaluation, various studies [13, 60], have established that it is not an effective means for evaluating the interpretability of extracted topics. Instead, Lau et et al. [42] demonstrated that the normalized pointwise mutual information (NPMI) coherence between word pairs in each topic closely aligns with human annotators’ evaluation of topic interpretability. Therefore, following the approach taken by [72], we use NPMI rather than perplexity as the primary evaluation metric.

To assess the quality of the topics extracted by each model, we adopt two coherence measures: $C_{NPMI}$ [1, 34] and $C_{V}$ [68]. This last measure combines the indirect cosine measure with the $C_{NPMI}$ and the boolean sliding window. Both measures have been shown to correlate with human judgments of topic quality with $C_V$ having the strongest correlation [68]. Even though $C_V$ has stronger correlation that $C_{NPMI}$ with human evaluations, $C_{NPMI}$ is more commonly used in the literature [34], possibly due to the extra computation required by $C_V$. We prefer the $C_V$ measure as, in addition to being more highly correlated with human judgment, it considers the similarity of the contexts of the terms, not just their own co-occurrence. We use Gensim^12^ to compute both measures and consider the top 5 terms of each topic for evaluation. Each dataset has a train/test split. We train all models on the train documents and evaluate using the test documents. We use the standard 110-term window for $C_V$ and 10-term window for $C_{NPMI}$. We use the top 5 terms of each topic for evaluation

#### Topic Diversity Measures

In addition to coherence measures, we also consider diversity metrics to assess the quality of topics produced by each model. These metrics are computed based on the distribution of topic words and provide a numerical score that indicates how diverse the words are in the topics. Ideally, for topics that are semantically different from each other, we expect the diversity scores to be close to 1. This is because diverse topics are more informative and useful for downstream applications such as document classification or information retrieval. In our experiments, we consider *PUW*, *PJD*, *IRBO* and, use implementation of topic diversity^13^ given by [75].

- **Proportion of Unique Words (PUW)** [19] is used to determine the percentage of unique words in a topic. A PUW score that is close to 0 indicates that the topic contains a lot of redundant words, while a score close to 1 suggests that the topic is more diverse and contains a wider variety of words.
- **The Average Pairwise Jaccard Diversity (PJD)** [78] measures the average pairwise Jaccard distance between the topics. The resulting diversity score increases as the topics become more dissimilar, providing better coverage of various aspects.
- **Inverted Rank-Biased Overlap (IRBO)** metric [4] is a measure of the rank-biased overlap between topics, indicating the diversity of topics generated by a single model. To calculate IRBO, we use the inverse of the standard RBO [76], which compares the top 10 words of two topics. The RBO^14^ metric allows for the possibility of disjointedness between the lists of topics, meaning that two topics can have different words, and uses weighted ranking. For instance, if two lists share some of the same words, albeit at different rankings, they are penalized less than two lists that share the same words at the highest ranks. An IRBO score of 0 indicates identical topics, while a score of 1 indicates completely different topics [85].

We believe that the combination of coherence and diversity metrics provides a more comprehensive evaluation of topic models and can help researchers to make informed decisions about which models to use for their specific applications.

#### Hierarchical Analysis

To measure the quality of the topic hierarchy, we use two measures proposed in [37]: topic specialization and hierarchical affinity.

- **Topic Specialization** measures the distance of a topic’s probability distribution over terms from the general probability distribution of all terms in the corpus given by their occurrence frequency. We expect topics at higher levels in the hierarchy closer to the root to be more general and less specialized and topics further down the hierarchy to be more specialized.
- **Hierarchical Affinity** measures the similarity between a super-topic and a set of sub-topics. We expect higher affinity between a parent topic and its children and lower affinity between a parent topic and sub-topics which are not its children.

### Comparative Baselines

#### Flat Topic Detection

Regarding the detection of flat topics, we evaluate our Community Topic algorithm against LDA [7], Top2Vec [2], an algorithm based on word embeddings learned by a neural network and BERTopic [30], which is similar to Top2Vec in terms of algorithmic structure but dedicated to topic detection. Another baseline we consider is Correlation Explanation (CorEx) [27], which employs an information-theoretic approach to learn latent topics over documents. Unlike LDA, CorEx does not make any assumptions about the data generating model and searches for topics that provide maximum information about a set of documents. We assess the performance of these algorithms in terms of topic coherence, diversity, runtime, and stability of topic quality across multiple runs.

We used the best hyper-parameters for CT to achieve the best evaluation metrics. For CT, we applied noun-only filtering and constructed co-occurrence networks using a sentence co-occurrence window and NMPI edge weights. We kept the edge weights as is, without applying any threshold for the noun-only corpus. For LDA and Top2Vec, we used noun-only POS filtering for 5 topics since 5 topics is the average number of flat topics obtained from community mining. We did not need to tune any hyperparameters for the Top2Vec algorithm. To run BERTopic, we provided the raw text corpus to the model and set the verbose flag to True, which helped to track the stages of the model. We then fit the BERTopic model on a collection of documents, generated topics, and returned the docs with topics. For CorEx, the topic model assumes that the input is in the form of a doc-word matrix, where rows represent documents and columns represent binary counts. Hence, we converted the raw data into the necessary format. We also set 6 different parameters for CorEx. To compare the run times and stability of these algorithms over repeated runs, we ran each algorithm 10 times. As the scores were almost similar, deviation was less and the results reported correspond to the best ones.

#### Hierarchical Topic Detection

Three probabilistic graphical topic models, namely HLDA [29], PAM [45], and HPA^15^ [55] serve as our hierarchical baselines.

HLDA can produce topics at three levels, which are probability distributions over vocabulary terms, and thus, they are compatible with our evaluation metrics without any modifications. On the other hand, CT generates a list of terms sorted by internal weighted degree, which we convert into probability distributions to calculate specialization and affinity by dividing each value by the sum of all values. The super-topics discovered by PAM and HPA are distributions over sub-topics. We convert into distributions over terms by computing the expectation for each term in the sub-topics given the super-topic distribution over sub-topics. However, since the super-topic distribution assigns a non-zero probability to all sub-topics, we need to distinguish between children and non-children. To address this, we consider the top six most likely sub-topics as the children of a super-topic, as we hypothesize an average of six sub-topics per super-topic in a topic hierarchy.

CT applies a Leiden resolution parameter of 1.0 to identify 5 or 6 super-topics across all datasets, each consisting of 5, 6, or 7 sub-topics on average, which serves as a guide for the PAM and HPA models. On the other hand, HLDA discovers hundreds of super-topics and roughly three times more sub-topics than CT. However, this approach of generating numerous small topics at all levels often leads to suboptimal results according to our evaluation metrics and an imperfect hierarchy, where a child topic is frequently present in more documents than its parent.

In addition, we compare CT to nTSNTM model [15], which leverages the neural variational inference (NVI) framework and a nonparametric prior to group topics into a sensible tree structure. We utilized the publicly available code of nTSNTM^16^ with the recommended parameters indicated in [15]. The model was trained for 100 epochs, with a hidden size of 256, and we ensured that it was compatible with the latest version of Tensorflow in order to obtain accurate results. To maintain consistency in hardware, we executed the nTSNTM model on the same commodity hardware used by the baseline models mentioned earlier. However, it should be mention that nTSNTM requires specific pre-processed data. But since the preprocessed data are only available for NG20 and Reuters, the experiments could only be carried out on these datasets. Moreover, as nTSNTM does not provide topic words, only evaluation measures computable from the produced results are reported.

## Experimental Results

### Results for Flat Topic Detection

*Topic coherence and diversity analysis* This first set of experiments allows to compare Community Topic (CT) with other popular topic modeling algorithms, namely LDA, Top2Vec, BERTopic, and CorEx for flat topics discovery.

**Table 1:** Best evaluation scores obtained on the datasets for flat topic detection

Models	Datasets	$C_{V}$	$C_{NPMI}$	PUW	PJD	IRBO	Time (seconds)
CT	BBC	**0**.**700**	**0**.**170**	**1**	**1**	**1**	**2**.**786**
	NG20	0.769	**0**.**166**	**1**	**1**	**1**	5.060
	Reuters	0.690	0.107	**1**	**1**	**1**	**4**.**051**
	EuroParl	0.535	0.044	**1**	**1**	**1**	**1**.**384**
LDA	BBC	0.461	−0.028	0.460	0.605	0.353	6.49
	NG20	0.552	0.038	0.800	0.9133	0.8663	**4**.**53**
	Reuters	0.453	0.002	0.620	0.796	0.580	5.32
	EuroParl	0.463	−0.009	0.860	0.957	0.927	3.990
Top2Vec	BBC	0.630	0.043	**1**	**1**	**1**	16.98
	NG20	0.655	0.082	0.637	0.966	0.998	62.47
	Reuters	0.686	0.158	0.473	0.923	0.996	55.53
	EuroParl	0.285	−0.482	**1**	**1**	**1**	92.71
BerTopic	BBC	0.550	0.041	0.504	0.767	0.843	309.592
	NG20	**0**.**784**	0.165	0.795	0.997	0.998	1436.311
	Reuters	**0**.**823**	**0**.**250**	0.682	0.997	0.998	1620.018
	EuroParl	**0**.**75**	**0**.**128**	0.746	0.998	0.998	473.070
CorEx	BBC	0.603	−0.023	**1**	**1**	**1**	62.634
	NG20	0.518	0.032	**1**	**1**	**1**	65.580
	Reuters	0.605	0.051	**1**	**1**	**1**	64.695
	EuroParl	0.314	−0.172	**1**	**1**	**1**	39.441

The best results are in bold

Table 1 presents a clear picture of the topic coherence and diversity scores obtained with these algorithms. Community Topic (CT) emerges as the most coherent algorithm in terms of $C_{V}$ and $C_{NPMI}$ among all, except BERTopic. Although Top2Vec produces more coherent topics than LDA and CorEx, it falls short of the coherence scores achieved by CT. Moreover, Top2Vec takes significantly longer and is less stable over repeated runs, making it less favorable for practical applications.

Both Top2Vec and BERTopic are word embedding-based models learned by a neural network, and our analysis shows that their coherence validation ($C_{V}$) scores are in general higher than other baselines.

However, both models fail to provide diverse topics, as indicated by the low scores for the diversity measures Proportion of Unique Words (PUW), Average Pairwise Jaccard Diversity (PJD), and Inverted Rank-Biased Overlap (IRBO). On the other hand, CT and CorEx stand out for their diverse topics, with CorEx producing the most diverse topics among all the baselines. However, CorEx lags behind CT in terms of $C_{NPMI}$ and $C_{V}$ scores.

*Run Time Analysis* Concerning the run time, our experiments showed that LDA, Top2Vec, BERTopic and Corex have more run times compare to CT. For CT the reported time combines the time for building network, applying community detection algorithm and the filtering/ordering task. It is important to note that the community detection algorithms used by CT can be significantly impacted by the size of the network. For larger networks, the run times of the algorithms can increase by about one order of magnitude, which is equivalent to half a second. Despite this, the network creation and topic filtering/ordering steps of CT remain the same for both smaller and larger networks. In terms of run times for the individual algorithms, CT has an average of 3 s, LDA takes 5 s, Top2Vec takes 56 s, BERTopic takes 960 s, and CorEx takes 58 s. While LDA and CT are faster compared to the other baselines, CT still emerges as the fastest of all, demonstrating its efficiency in processing large datasets and its potential usefulness in real-world applications.

Overall, the evaluation metrics reveal that each algorithm has its own strengths and weaknesses, and the choice of an appropriate algorithm depends on the specific requirements of the project. CT and BERTopic offer high coherence. Community Topic (CT) appears a suitable option since it considers all these factors and strives to produce high-quality topics.

**Fig. 3:**
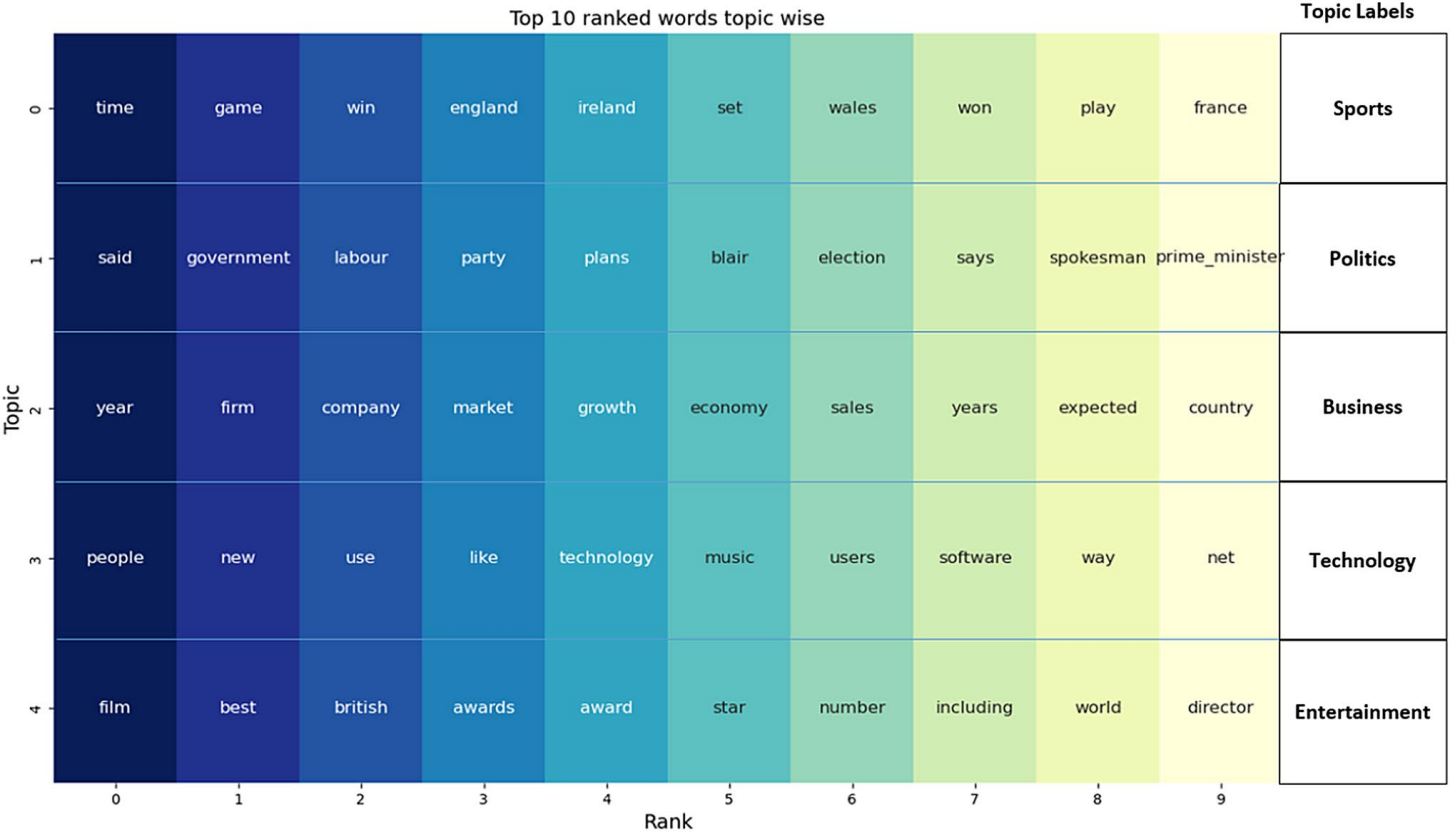
Top 10 words per topic produced by CT on BBC corpus

*Qualitative evaluation of the extracted topics* In addition, we also compared the top 10 terms produced by CT and LDA on the BBC. To achieve this, CT utilized Leiden with a resolution parameter of 1.0, sentence co-occurrence, NPMI edge weights, and no thresholding to discover five topics. As shown in Fig. 1, the top 10 terms in each of the discovered topics were found to be coherent, diverse, and unique, representing the categories of “Politics," “Technology," “Business," “Sports," and “Entertainment." The ranking of the top 10 words was based on internal degree weight in the community, which was described in the methodology section (Fig. 3).

In contrast, the topics generated by LDA, are less natural and tend to have overlapping content as as shown in Table 2 which presents the top 10 words produced by LDA on BBC corpus. Notably, we can observe that several words, including year, people, government, time, film, and game, are present in multiple topics. Consequently, the topic diversity is undermined, resulting in less distinctive and unique topics.

**Table 2:** Top 10 words per topic produced by LDA on BBC corpus

Topics	
**year**, *people*, time, ***world***, **years**, game, *government*, technology, music, way	
*People*, **year**, time, film, *government*, ***world***, number, way, game, **year**	
**year**, company, firm, **years**, *government*, week, economy, *people*, growth, ***world***	
**Year**, *people*, time, game, film, ***world***, **years**, number, club, wales	
**year**, *people*, *government*, time, election, labor, **years**, party, plans, music	

The best results are in bold

Thus, based on our analysis, CT is able to produce non-overlapping topics, resulting in clear and distinct topic boundaries in documents. Moreover, it achieves this with the fastest processing times compared to other algorithms. The added advantage of being able to run CT on commodity hardware further adds to its appeal. Additionally, CT produces highly coherent topics, which makes it more user-friendly and easier to interpret.

### Results for Topic Hierarchy Detection

**Table 3:** Best evaluation scores obtained on the datasets for hierarchical topics

Model	Coherence	BBC	NG20	Reuters	EuroParl
CT	$C_{V}$	**0**.**661**	**0**.**753**	**0**.**709**	0.420
	$C_{\textrm{NMPI}}$	0.075	**0**.**132**	**0**.**166**	−0.139
HLDA	$C_{V}$	0.432	0.428	0.447	0.327
	$C_{\textrm{NMPI}}$	**0**.**187**	−0.146	−0.102	−0.269
PAM	$C_{V}$	0.595	0.652	0.640	**0**.**480**
	$C_{\textrm{NMPI}}$	0.059	0.114	0.091	−**0.021**
HPA	$C_{V}$	0.614	0.632	0.627	0.439
	$C_{\textrm{NMPI}}$	0.069	0.088	0.096	$-$0.080

The best results are in bold

*Topic coherence comparison with parametric models* Concerning topic hierarchy detection, Table 3 presents the coherence scores $C_V$ and $C_{NMPI}$ for CT, HLDA, PAM and HPA. They show that CT outperforms other algorithms in terms of coherence score $C_V$ on all datasets, except for EuroaParl, where PAM achieves the highest score followed by HPA. In contrast, HLDA obtains the lowest score, indicating that the topics generated by CT are more interpretable to human users.

The consistency in topics found by CT across multiple datasets is promising, and the high coherence scores suggest that the topics identified by CT are highly interpretable. These findings could be useful for researchers and practitioners who use topic modeling to analyze large datasets and extract meaningful insights from them.

*Run time comparison with parametric models* Moreover, out of all the algorithms, CT is the most efficient, taking less than 5 s to discover the topic hierarchy on all datasets. On the other hand, HLDA requires between 30 s to 5 min, while PAM and HPA range from 10 s to 2 min. It’s worth noting that all experiments were conducted on a laptop with a 2.7 GHz dual-core processor and 8 GB RAM, ensuring a fair comparison between the algorithms.

*Comparison with non parametric model nTSNTM*

As part of our experiments, we incorporated the Tree-Structured Neural Topic Model (nTSNTM) that employs non-parametric neural variational inference.

**Table 4:** Scores obtained by CT and nTSNTM

Model	Dataset	$C_{NMPI}$	PUW	Time (seconds)
CT	NG20	0.132	**0**.**871**	**4**.**95**
	Reuters	0.166	**0**.**862**	**13**.**67**
nTSNTM	NG20	**0**.**242**	0.757	11700
	Reuters	**0**.**240**	0.661	7380

The best results are in bold

Table 4 presents the scores obtained by CT and nTSNTM on NG20 and Reuters datasets. The results indicate that while nTSNTM outperforms CT in terms of $C_{NPMI}$ score, CT performs better in terms of topic diversity. Moreover, nTSNTM takes on average, a total time of three hours to run on commodity hardware, while CT completes the same task in just a few seconds.

**Fig. 4:**
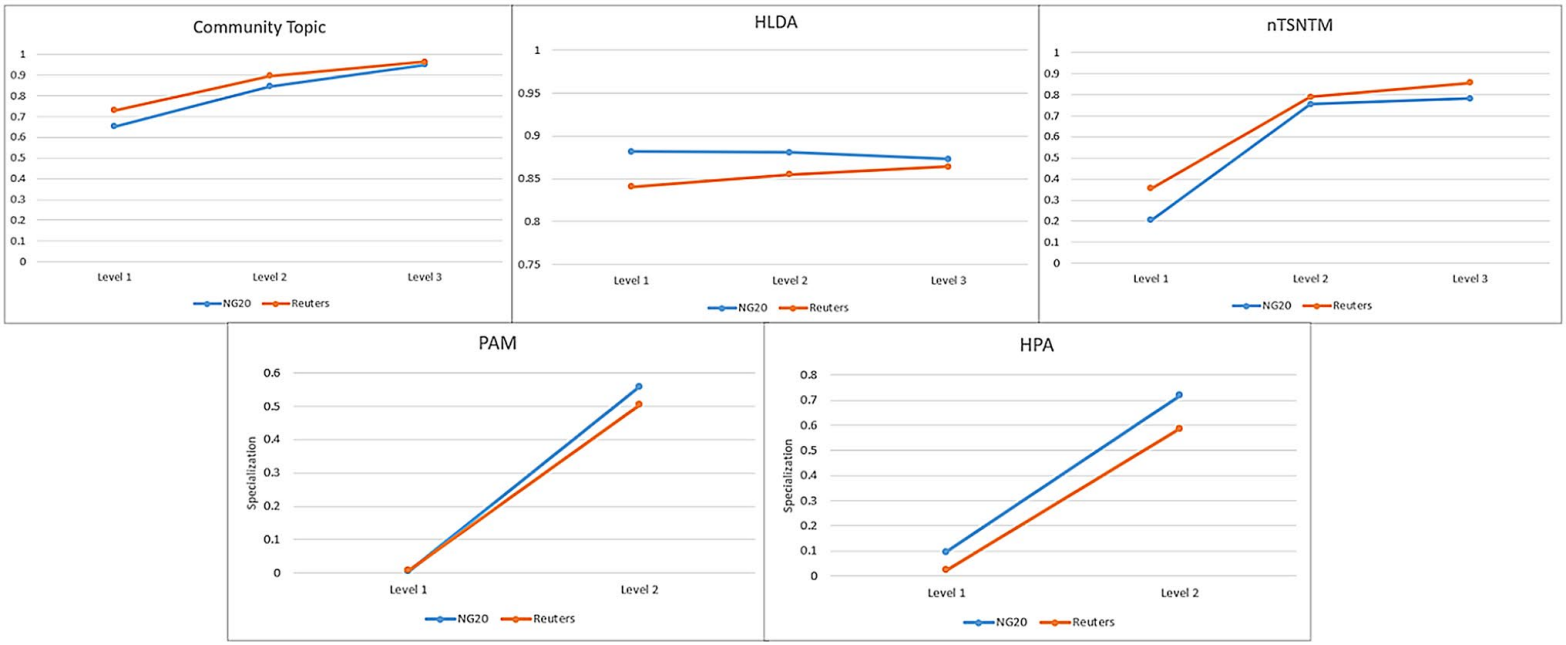
Specialization Scores obtained on NG20 and Reuters

*Topic specialization analysis* As indicated in [81], an effective topic hierarchy is characterized by topics at the top being more general and those at the bottom being more specific. Figure 4 illustrates the specialization scores for each algorithm on the NG20 and Reuters Datasets. We observed that CT, HLDA, and nTSNTM found both super-topics (level 1), sub-topics (level 2), and sub-topics of subtopics (level 3), while PAM and HPA only supported super-topics and sub-topic hierarchies. HLDA has a very high specialization score, consistent with the large number of topics found at all three levels, but it does not align with our intuition that higher-level topics should be more general. PAM produces general topics at level 1 and more specialized topics at level 2, but the super-topics are too general and similar to the overall frequency distribution to provide useful information for the user. HPA produces a similar level of specialization as PAM, except that it generates slightly more specialized topics for NG20 at level 1, but not more than CT. nTSNTM shows an increasing specialization from level 1 to level 3, with more specialized topics at level 1 than PAM and HPA. However, CT outperforms all of the models by producing reasonably high specialization for level 1 that increases up to level 3.

**Fig. 5:**
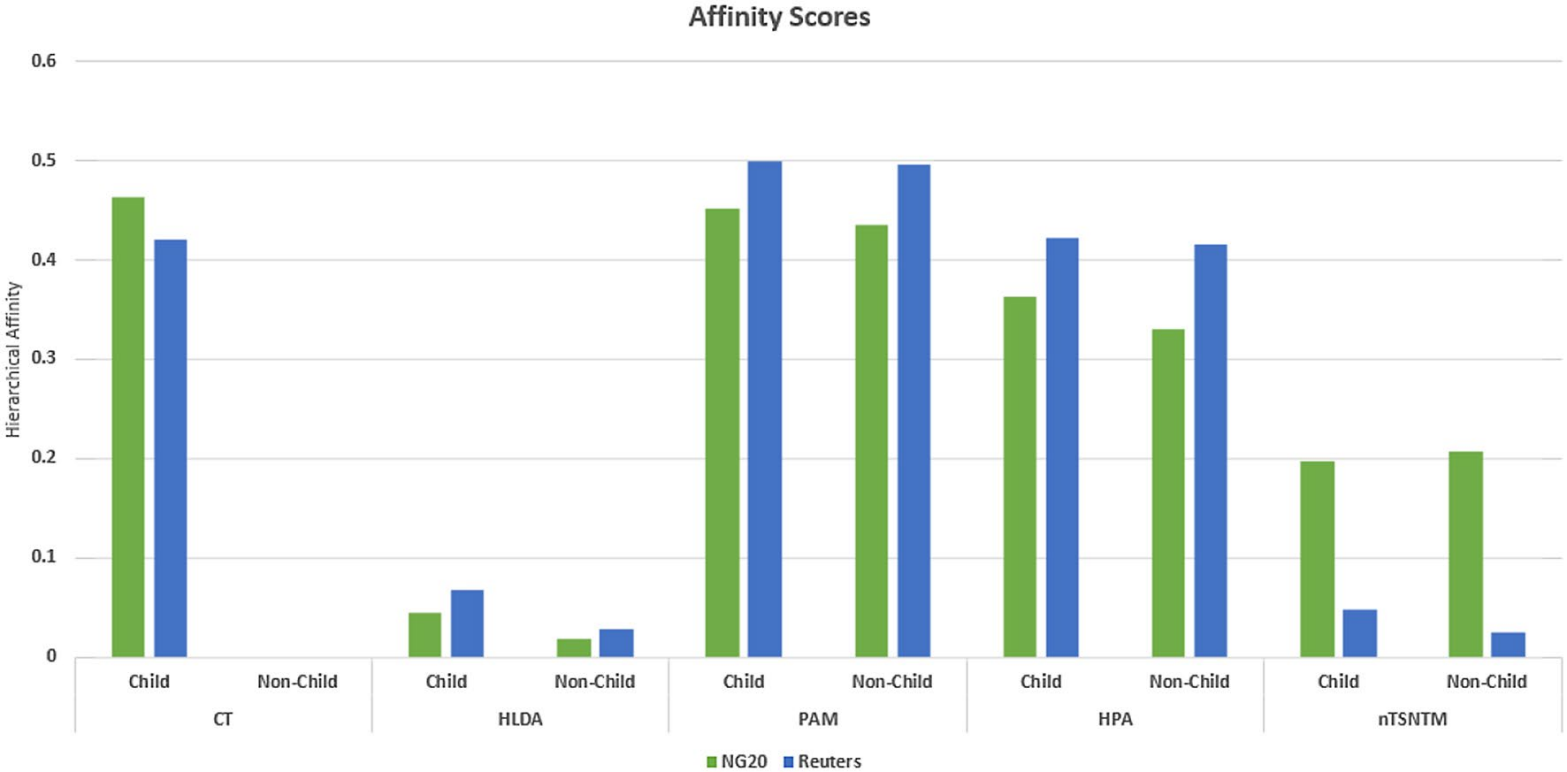
Affinity scores obtained on NG20 and Reuters

The hierarchical affinity scores of each algorithm on the NG20 and Reuters datasets are presented in Fig. 5. It can be observed that HLDA displays a higher affinity between parent topics and their children, but the overall affinity is very low, leading to a weak relationship between super-topics and sub-topics. On the other hand, HPA and PAM exhibit high affinities between parent topics and both child and non-child topics, as their super-topics are distributions over all sub-topics and are thus non-specialized. In contrast, CT parent topics demonstrate high affinity with their children and no affinity with non-children since the sub-topics are a partition of the super-topic and do not overlap with any other super-topic. For nTSNTM, the affinity between parent topics and their children is almost the same as non-children for NG20, and slightly better for Reuters. This indicates that nTSNTM does not produce a strong linkage between parents and their children, which contradicts its higher $C_{NPMI}$ score compared to other models.

**Fig. 6:**
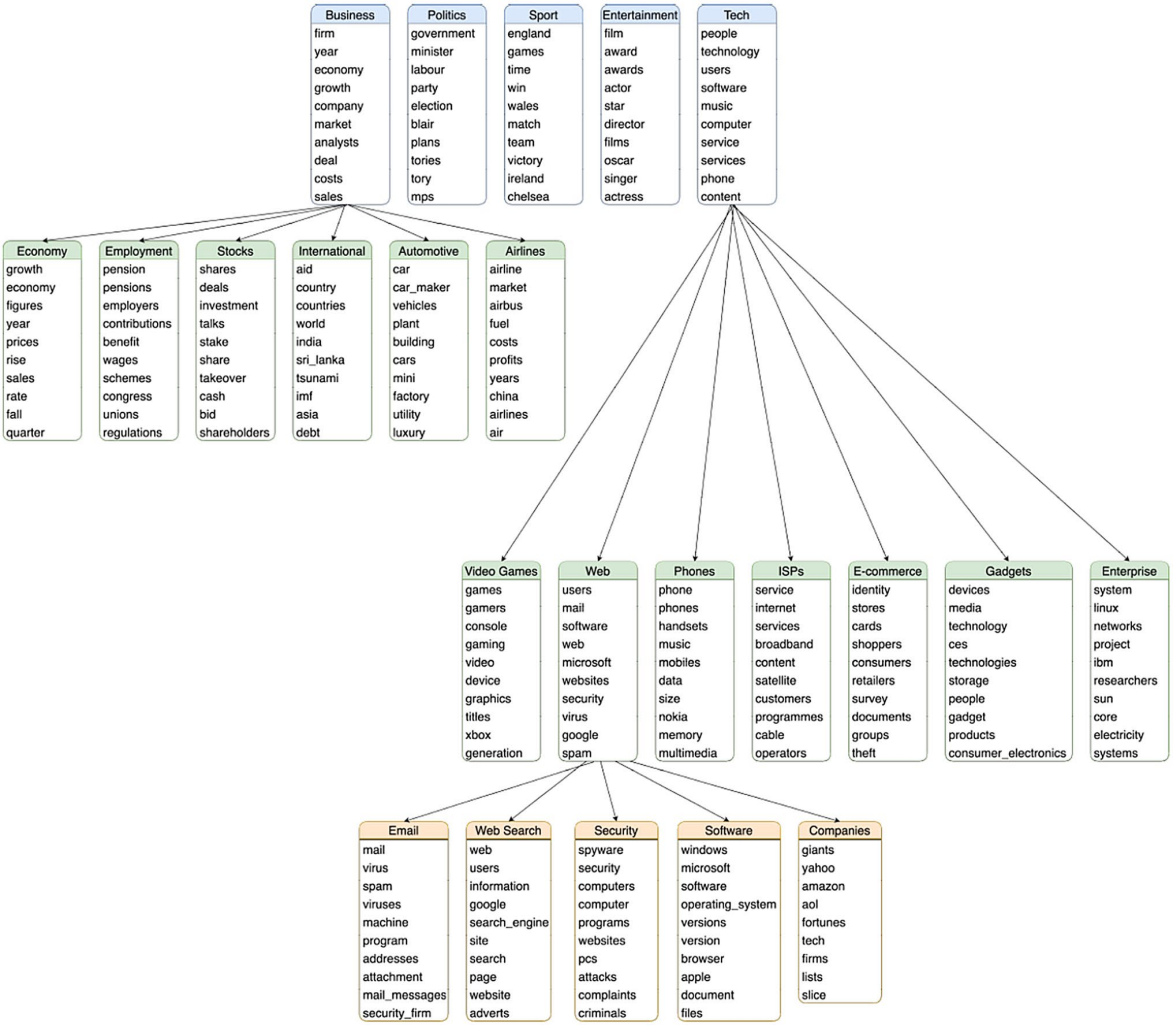
Hierarchy of BBC corpus topics found by iteratively applying CT algorithm

**Fig. 7:**
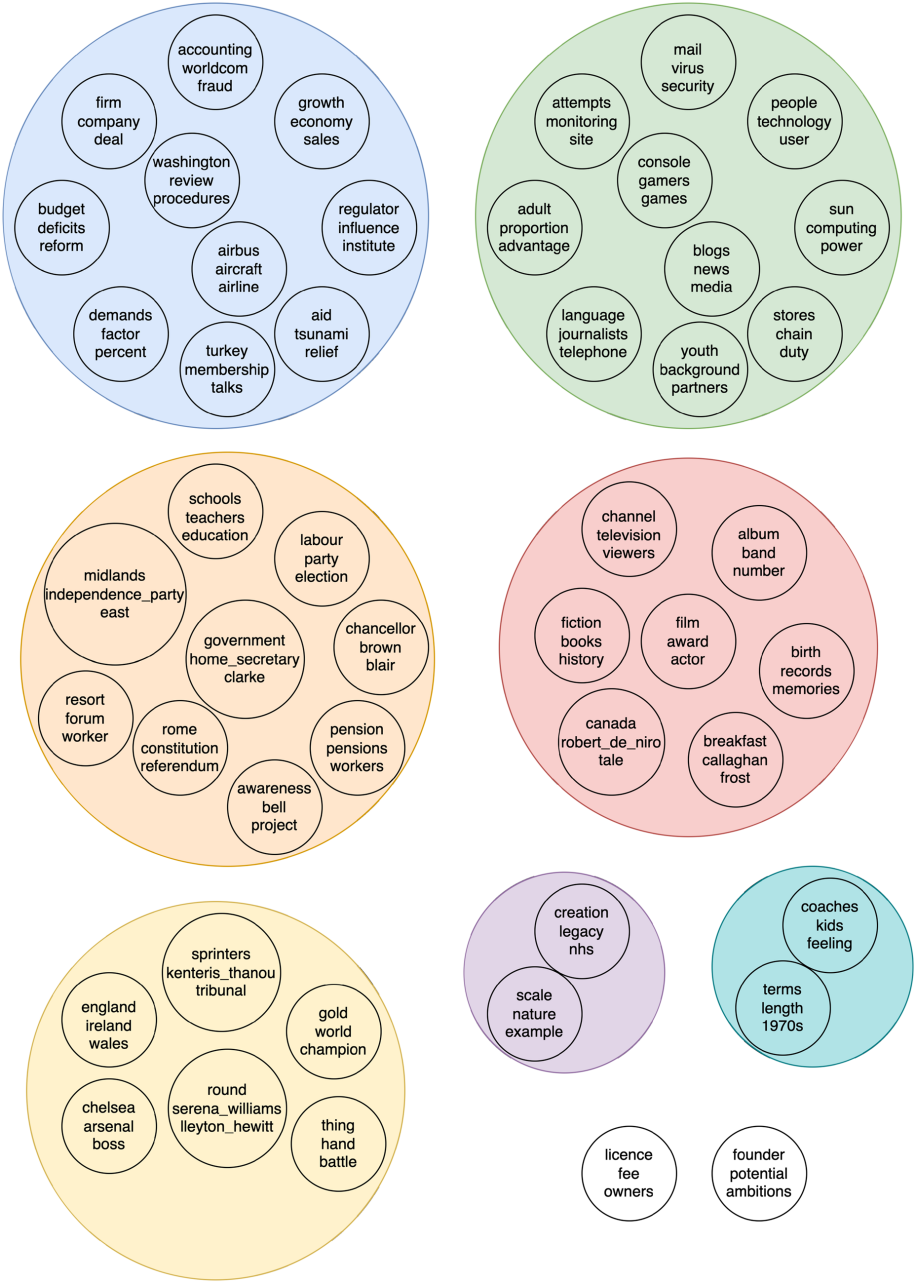
Super-topics found by applying community detection on network of small topics

For illustration, an example of 3 levels of topics discovered by CT on the BBC corpus is show in Fig. 6. The level 1 topics correspond to the 5 article categories of the corpus. Level 2 and then 3 show increasingly specific sub-topics. Applying CT with Leiden again to the “Tech” topic finds 7 sub-topics such as “video games”, “the web”, and “cellphones”. “The web” sub-topic produces another set of 5 sub-sub-topics such as “email”, “web search”, and “internet security”. With a resolution parameter of 2, CT with Leiden initially finds a set of 48 small topics. Performing community detection on the network of topics results in 9 super-topics, 5 of which are large and correspond to the article categories. These super-topics are shown in Fig. 7.

After evaluating the performances of CT, we have come to the conclusion that CT with Leiden is the most effective one. It offers the most comprehensive topic hierarchy, which can cater to communities of varying sizes, and performs consistently well across all datasets using the same CT hyperparameters. Moreover, CT with Leiden is incredibly fast and can generate a coherent topic structure in a shorter duration than other algorithms, even when using commodity hardware.

Our experiment findings reveal that CT generates clear and interpretable topics with the best hierarchy. The topic hierarchy produced by CT demonstrates greater specialization for sub-topics as compared to super-topics, while still maintaining enough specificity at both levels to make the topics useful. Furthermore, the super-topics of CT show a strong affinity with their corresponding sub-topics, indicating a robust linkage.

### Evaluation of CT on Different Languages

CT is a graph-based method which exploits the co-occurrences of the words. As co-occurrence is valid independently of the language, CT is not specific to English but it is language agnostic. Therefore it can work with any language. In order to further explore this capability of CT, we conducted experiments on documents written in five different languages: English, Italian, French, German, and Spanish. The baselines for these experiments were the same as that used for the flat topic experiments. Though, the BERTopic baseline failed to run on French language for which CamemBERT is most suited [50]. We chose the EuroParl dataset as it provides the same content in different languages, making it ideal for measuring the consistency of the algorithm across languages.

**Table 5:** Evaluation scores obtained on EuroParl dataset across different languages

Model	Language	$C_{V}$	$C_{NPMI}$	PUW	PJD	IRBO
CT	English	0.535	0.043	**1**	**1**	**1**
	Italian	0.555	0.036	**1**	**1**	**1**
	French	0.554	0.033	**1**	**1**	**1**
	German	0.534	**0**.**009**	**1**	**1**	**1**
	Spanish	**0**.**579**	0.051	**1**	**1**	**1**
LDA	English	0.411	−0.065	0.88	0.951	0.929
	Italian	0.543	−0.021	0.480	0.641	0.227
	French	**0**.**567**	−0.013	0.400	0.470	0.768
	German	**0**.**571**	−0.018	0.440	0.603	0.527
	Spanish	0.557	−0.004	0.440	0.615	0.437
Top2Vec	English	0.268	−0.215	**1**	**1**	**1**
	Italian	0.320	−0.491	0.453	0.943	0.977
	French	0.555	**0**.**036**	0.614	0.922	0.942
	German	0.316	−0.496	0.500	0.882	0.911
	Spanish	0.264	**0**.**491**	**1**	**1**	**1**
BerTopic	English	**0**.**750**	**0**.**142**	0.735	0.998	0.998
	Italian	**0**.**736**	**0**.**104**	0.819	0.999	0.999
	German	0.407	−0.104	0.853	0.998	0.999
	Spanish	0.330	−0.147	0.884	0.999	0.999
CorEx	English	0.314	−0.172	**1**	**1**	**1**
	Italian	0.385	−0.157	**1**	**1**	**1**
	French	0.419	−0.175	**1**	**1**	**1**
	German	0.452	−0.059	**1**	**1**	**1**
	Spanish	0.354	−0.161	**1**	**1**	**1**

The best results are in bold

The results in terms of coherence and diversity are presented in Table 5. CT performs better or equivalent to Top2Vec and CorEx for all languages in terms of coherence scores ($C_{V}$ and $C_{NPMI}$), as seen previously for flat topic detection. BERTopic achieves the highest coherence scores, but it is worth noting that CT exhibits consistency across different languages for the same dataset, with scores ranging from 0.530 to 0.580. In contrast, BERTopic has high scores for English and Italian but experiences a decline of around 30% for Spanish. Although LDA produces good scores for the French, Spanish and German languages compared to CT, it has negative $C_{NPMI}$ scores. Overall, CT yields consistent and positive $C_{NPMI}$ coherence scores for all languages.

The topic diversity for CT and CorEx equals 1 across all languages. However, BERTopic and LDA show poor diversity across all languages. Top2Vec produces more diverse topics for English and Spanish, but fails to maintain this diversity for Italian and German. Furthermore, the time taken by all the algorithms remains the same as in the flat topic experiments, with CT remaining the fastest algorithm.

**Fig. 8:**
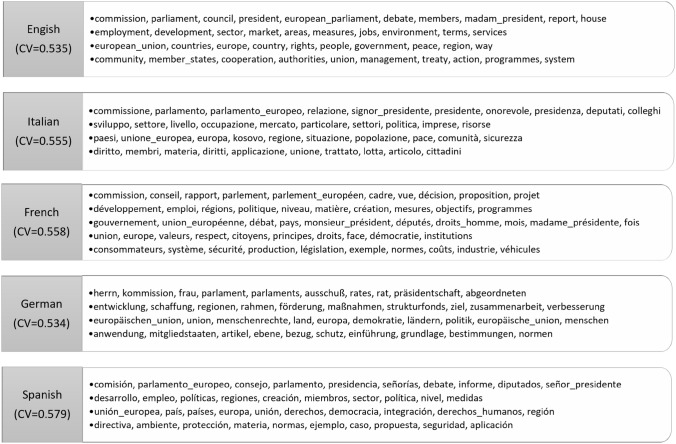
Top 10 words per topic for different languages on EuroParl dataset

To showcase the human interpretability of the topics generated by our approach, we have leveraged DeepL translation^17^ to translate the resulting topics into English. We observed that the translated topics have similar themes across languages. Furthermore, Fig. 8 displays the top 10 words of each topic generated by our method, after translation. Notably, CT produces consistent topics, with diversity and coherence maintained for all languages, which demonstrates its consistency and robustness.

## Conclusion

This paper presents a novel topic modeling algorithm, Community Topic (CT), that combines the fields of topic modeling and social network analysis to overcome the deficiencies of existing popular approaches.

We believe that graph-based topic modeling allows to approach topic discovery from a new angle that does not require specifying the underlying distributions unlike Bayesian models. This makes it possible to find topics of different sizes. On the other hand, it supposes an adequate extraction of the words from the documents in order to control the size of the graph and consequently the processing times.

Our experiments show that CT outperforms other popular algorithms in terms of coherence, topic diversity, and interpretability. The results also indicate that CT remains consistent across different languages with similar dataset content and thus can potentially aid in various natural language processing tasks. It also provides a topic structure that can be utilized in downstream tasks since sub- and super-topics can be found and there are relationships between topics which can all be used to guide a researcher exploring a corpus or an agent having a conversation.

Looking ahead, there are several avenues for further research to enhance the quality of topics generated on co-occurrence networks.

A first perspective relies in the extension of CT to allow for overlapping topics. Currently, topics are partitions of the vocabulary, but introducing a method such as persona splitting [24] could create multiple instances of a vertex and enable terms to fall into multiple topics. Another option consists to apply a method for overlapping community detection [82] instead of Leiden. Indeed, whereas classical community detection methods assume the division of nodes as a partition problem and thus restrict a node to belonging to only one community, with overlapping community detection approaches, a node can be part of multiple groups simultaneously. This is particularly interesting in the context of topic detection by community discovery on word co-occurrence network since, by this way, a word could belong to two different topics. For example, the word "Jaguar" could appear in two communities depending on its meaning, the first one containing terms linked to animals and in particular to panthers, the second to cars. Thus, overlapping community detection into CT, makes it possible to deal, very simply, with the case of polysemy. This would open up new possibilities for more nuanced and granular topic modeling, and could enhance the practical applications of CT in domains such as information retrieval and natural language processing.

Additionally, we plan to investigate the effectiveness of CT on short-text data, such as sentences, and optimize its performance in this context.

Finally, another possible direction for future exploration relies in the exploitation of our topic model in concrete application. Indeed, if automated coherence metrics can provide some insight into the quality of topics, we aim to take this a step further by integrating CT into a conversational agent and testing the coherence and structure of topics in a real-world application.

## Footnotes

1 https://github.com/DATAMI01/DSA.

2 https://github.com/DATAMI01/DSA.

3 https://shr1911.github.io/communitytopic/api-reference/.

4 https://www.kaggle.com/competitions/learn-ai-bbc/data.

5 https://github.com/DATAMI01/DSA

6 https://scikit-learn.org/0.19/datasets/twenty_newsgroups.html

7 https://huggingface.co/datasets/reuters21578

8 https://www.kaggle.com/competitions/learn-ai-bbc/data

9 https://www.statmt.org/europarl/

10 https://spacy.io/

11 https://radimrehurek.com/gensim/.

12 https://radimrehurek.com/gensim/models/coherencemodel.html.

13 https://github.com/MIND-Lab/OCTIS.

14 https://github.com/dlukes/rbo.

15 https://bab2min.github.io/tomotopy/v0.12.2/en/.

16 https://github.com/hostnlp/nTSNTM.

17 https://www.deepl.com/translator.
